# Identification and Validation of a Core Single-Nucleotide Polymorphism Marker Set for Genetic Diversity Assessment, Fingerprinting Identification, and Core Collection Development in Bottle Gourd

**DOI:** 10.3389/fpls.2021.747940

**Published:** 2021-11-18

**Authors:** Ying Wang, Xiaohua Wu, Yanwei Li, Zishan Feng, Zihan Mu, Jiang Wang, Xinyi Wu, Baogen Wang, Zhongfu Lu, Guojing Li

**Affiliations:** ^1^Institute of Vegetables, Zhejiang Academy of Agricultural Sciences, Hangzhou, China; ^2^State Key Laboratory for Managing Biotic and Chemical Threats to the Quality and Safety of Agro-Products, Zhejiang Academy of Agricultural Sciences, Hangzhou, China

**Keywords:** bottle gourd, SNP, germplasm collections, fingerprint, core population

## Abstract

Germplasm collections are indispensable resources for the mining of important genes and variety improvement. To preserve and utilize germplasm collections in bottle gourd, we identified and validated a highly informative core single-nucleotide polymorphism (SNP) marker set from 1,100 SNPs. This marker set consisted of 22 uniformly distributed core SNPs with abundant polymorphisms, which were established to have strong representativeness and discriminatory power based on analyses of 206 bottle gourd germplasm collections and a multiparent advanced generation inter-cross (MAGIC) population. The core SNP markers were used to assess genetic diversity and population structure, and to fingerprint important accessions, which could provide an optimized procedure for seed authentication. Furthermore, using the core SNP marker set, we developed an accessible core population of 150 accessions that represents 100% of the genetic variation in bottle gourds. This core population will make an important contribution to the preservation and utilization of bottle gourd germplasm collections, cultivar identification, and marker-assisted breeding.

## Introduction

Bottle gourd [*Lagenaria siceraria* (Mol.) Standl.] (2n = 2x = 22), also known as calabash or long melon, is a cultivated vegetable, medicinal plant, decorative cucurbit, and grafting rootstock belonging to the Cucurbitaceae family, together with other major cucurbit crops, including cucumber (*Cucumis sativus* L.), melon (*Cucumis melo* L.), and watermelon (*Citrullus lanatus* L.) (Heiser, 1979; Beevy and Kuriachan, 1996; Erickson et al., 2005; Morimoto et al., 2005). Recently, a high-quality bottle gourd reference genome (contig N50 = 11.2 Mb, scaffold N50 = 28.4 Mb) of 297 Mb was released for the Chinese landrace “Hangzhou Gourd” (Xu et al., 2021).

Currently, an increasing number of homogeneous new bottle gourd varieties are being released onto the market, and intellectual property disputes could emerge in cases where different species have been assigned the same name or where different names have been assigned a single species. Traditional identification and registration of germplasm collections or varieties are primarily dependent on field planting, which is both time-consuming and inefficient. Moreover, plant traits are often influenced by environmental variation (Jamali et al., 2019). Therefore, a precise, rapid, convenient, and cost-effective procedure, e.g., using molecular markers to predict traits (Jones and Mackay, 2015), is urgently needed to resolve intellectual property disputes and for cultivar improvement.

Molecular DNA markers are being used in an increasing number of crops for seed authentication, genetic diversity analysis, DNA fingerprint construction, and core collection development (Lv et al., 2012; Zhang et al., 2012; Hao et al., 2016; Yang et al., 2019). Several molecular markers have been developed for bottle gourd, including random amplified polymorphic DNA (RAPD), simple sequence repeat (SSR), and insertion-deletion (InDel) markers (Morimoto et al., 2005; Xu et al., 2011; Sarao et al., 2014; Wu et al., 2017). In addition, 3,226 single-nucleotide polymorphisms (SNPs) were identified by restriction-site associated DNA sequencing (RAD-Seq) genotyping of a natural population, and it was suggested that two sub-gene pools (Sub R and Sub L) were associated with fruit shape (Xu et al., 2014). SNP markers are suitable for high-throughput genotyping, due to their unique characteristics, including wide distribution, high density, and good stability (Su et al., 2018; Wang et al., 2018; Li et al., 2019; Wu et al., 2021). In this regard, Kompetitive allele-specific PCR (KASP) is a flexible, user-friendly SNP genotyping system that has been used for SNP genotyping in wheat, common bean, *Brassica rapa*, and cowpea (Allen et al., 2010; Cortés et al., 2011; Su et al., 2018; Li et al., 2019; Wu et al., 2021). The selection and development of a core SNP marker set with a high-throughput SNP genotyping platform, which can be used for rapid assessment and fingerprinting of germplasm collections, is essential for marker-assisted breeding and cultivar identification in bottle gourd.

Although originating from Africa, the bottle gourd was in use by humans in east Asia, the Americas, Europe, and the South Pacific (Erickson et al., 2005; Schlumbaum and Vandorpe, 2012; Kistler et al., 2014). Bottle gourd populations exhibit a tremendous diversity in fruit shape (Heiser, 1979; Morimoto and Mvere, 2004; Xu et al., 2014, 2021), based on which the bottle gourd populations are consistently grouped rather than the geographical origin (Xu et al., 2011; Mladenovic et al., 2012; Yildiz et al., 2015). Bottle gourd germplasm is preserved in several seed banks and is used in various research by different institutions across the world (Morimoto et al., 2005; Achigan-Dako et al., 2008; Gurcan et al., 2015; Xu et al., 2021).

With regard to the preservation and utilization of germplasm collections in plant breeding, Frankel (1984) were the first to propose the concept of core collections. A core collection is a subset of the total germplasm collection that is designed to represent a substantial proportion of the overall genetic diversity of the collection as a whole (Brown, 1989). Subsequently, core collections have been established in a range of plants, including wheat (Balfourier et al., 2007), rice (Zhang et al., 2011), and barley (Muñoz-Amatriaín et al., 2014). In addition, publicly accessible core collections have been developed for cucurbit crop cucumbers with advanced genetic variation (Lv et al., 2012; Wang et al., 2018). However, to date, there have been no efforts to preserve and utilize core collections in bottle gourd germplasm collections.

In this study, based on the re-sequencing of 20 representative bottle gourds, we selected and identified a core SNP marker set consisting of 22 core SNPs with abundant polymorphisms evenly distributed throughout the bottle gourd genome. To demonstrate the representativeness of the 22 core SNPs, we evaluated the polymorphism and discrimination of the core SNP set using 206 bottle gourd collections and a multiparent advanced generation inter-cross (MAGIC) population. Using this core SNP marker set, we assessed the genetic diversity and population structure of bottle gourd collections, fingerprinted bottle gourd germplasm collections and commercial cultivars, performed an optimized procedure for seed authentication, and developed an accessible core population. The results thus obtained will play a vital role in the preservation and utilization of bottle gourd germplasm collections, as well as in cultivar identification.

## Materials and Methods

### Plant Materials and DNA Extraction

In this study, we utilized a total of 206 bottle gourd germplasm collections for core SNP marker set screening, all of which are inbred lines (Supplementary Table 1). Twenty bottle gourd germplasm collections with diverse agronomic traits and genetic backgrounds were selected as representatives and were employed to re-sequence the whole bottle gourd genome for SNP discovery and selection. A total of 377 elite lines of a bottle gourd MAGIC population (Supplementary Figure 1) were utilized to assess the potential polymorphism of the core SNP markers developed in our study.

All accessions were grown in a growth incubator at 28°C/22°C with a 16 h light/8 h dark regime, and for each accession, young leaves from three independent individuals were collected for genomic DNA extraction using the cetyltrimethylammonium bromide (CTAB) method (Maguire et al., 1994). DNA quality was verified by electrophoresis on a 0.8% agarose gel, and the DNA concentrations were quantified using a NanoDrop 2000 UV spectrophotometer (Thermo Fisher Scientific, Waltham, MA, United States) and adjusted to a concentration of 20 ng/μL with sterile water.

### Single-Nucleotide Polymorphism Discovery and Selection

As a reference for SNP discovery, we used the Chinese landrace bottle gourd “HZ gourd” (Xu et al., 2021). Based on the SNP polymorphism information content (PIC) values of the genotypes of the 20 bottle gourd representatives that were re-sequenced in a recently published study (Xu et al., 2021), we selected SNPs for identification by KASP assays on another 22 distantly related accessions that were selected from the 206 bottle gourd germplasm collections. After eliminating low-quality and low-discriminatory SNPs, high-quality SNPs were selected for core SNP marker screening of the 206 bottle gourd germplasm collections. Core SNP markers were examined using previously published protocols (Yang et al., 2019), based on the even distribution of SNPs per chromosome and the principle of a minimum number of SNPs representing the maximum genetic diversity.

### Kompetitive Allele-Specific PCR Genotyping

Kompetitive allele-specific PCR assays were carried out using a Bio-Rad CFX96 Touch q-PCR System (Bio-Rad, CA, United States) with KASP genotyping reaction mixtures that included a KASP assay mix (containing two 12 nmol/L allele-specific primers and 30 nmol/L common reverse primer), a KASP master mix (KBS-1016-011; KBioscience, Hoddesdon, United Kingdom), and DNA samples (20 ng/μL). The cycling program was as follows: 15 min at 94°C, 10 touchdown cycles of 94°C for 20 s and 61–55°C for 60 s (decreasing by 0.6°C per cycle), and 26 cycles of 94°C for 20 s and 55°C for 60 s. An Omega Fluorostar scanner (BMG Labtech, Ortenberg, Germany) was used for fluorescence detection of the reaction products. Kluster Caller 1.1 (KBioscience, Hoddesdon, United Kingdom) software was used for data analysis, and SNPviewer2 (KBioscience, Hoddesdon, United Kingdom) software was used to read the reaction plates. Detailed instructions can be found at www.kbioscience.co.uk.

### Data Analysis

PowerMarker software^1^ was used to calculate genetic diversity and the PIC. GenAlEx 6.5 (Peakall, 2012) was performed to estimate minor allele frequency (MAF) and observed heterozygosity. Tassel 5.1 (Bradbury et al., 2007) was used to perform principal component analysis (PCA), and MEGA 5 (Tamura et al., 2011) was used to construct a neighbor-joining (NJ) tree based on Nei’s standard genetic distance (Nei, 1978). STRUCTURE V2.3 was used to analyze population structure (Pritchard et al., 2000; Falush et al., 2003). STRUCTURE HARVESTER (Earl and Vonholdt, 2012) was used to determine the most likely *K* value based on the Δ*K* method (Evanno et al., 2005). A barcode online generator^2^ was used to convert each SNP fingerprint into a barcode.

### Core Collection Development

The selection of core collections was carried out using Core Hunter (Thachuk et al., 2009), an algorithm for sampling genetic resources based on multiple genetic measures. Meanwhile, using this Core Hunter software, the Shannon–Weaver diversity index (I), Nei’s gene diversity index (H), and PIC between the core and whole collections were calculated and compared.

## Results

### Selection of High-Quality Single-Nucleotide Polymorphisms

The re-sequencing of the 20 bottle gourd representatives (Xu et al., 2021) generated a total of 1,843,914 SNPs. After filtering based on the criteria of a minor allele frequency of >5% and missing data rate of <5%, we obtained 723,946 filtered SNPs. Based on the SNP PIC values and the distribution of the re-sequenced bottle gourds, 1,100 SNPs (100 SNPs per chromosome and as evenly distributed as possible) were finally selected for identification by KASP assays. Twenty-two distantly related bottle gourd germplasm collections from among the 206 assessed collections were used to screen high-quality SNPs from the 1,100 SNPs using KASP assays.

**FIGURE 1:**
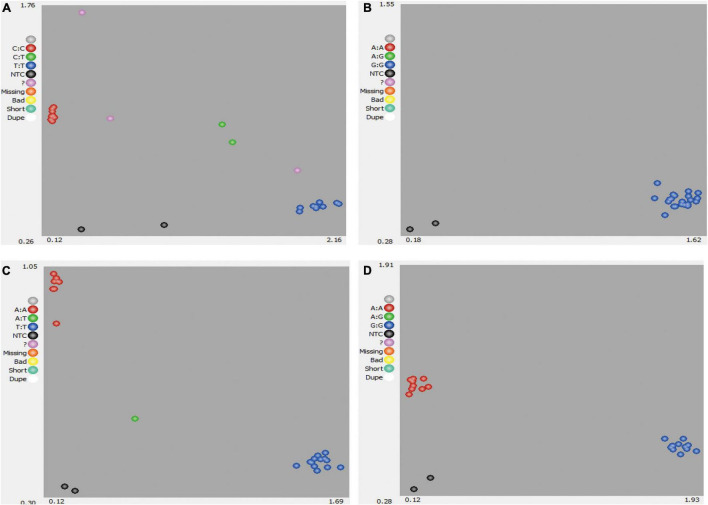
Development of SNP markers from 22 bottle gourd germplasm collection representatives for KASP genotyping. **(A)** A poorly amplified KASP marker. **(B)** A non-polymorphic KASP marker. **(C,D)** Strongly amplified KASP markers with high polymorphism. Red dots indicate samples homozygous for one allele, blue dots indicate samples homozygous for the second allele, and green dots indicate samples heterozygous for both alleles.

Single-nucleotide polymorphisms could be called for AA, BB, and AB genotypes (Figure 1). Where data points could not be clearly called for AA, BB, and AB genotypes (Figure 1A) or no polymorphism was identified (Figure 1B), these SNPs were deemed to be low-quality or low-discriminatory SNPs. For high-quality SNPs, discrimination between the two homozygous alleles (AA and BB) and heterozygous allele (AB) in the 22 bottle gourd germplasm collections was relatively straightforward (Figures 1C,D). Finally, 93 high-quality SNPs were selected and used to screen the 206 bottle gourd germplasm collections to identify potential core SNP marker sets.

### Identification of Candidate Core Single-Nucleotide Polymorphisms

**FIGURE 2:**
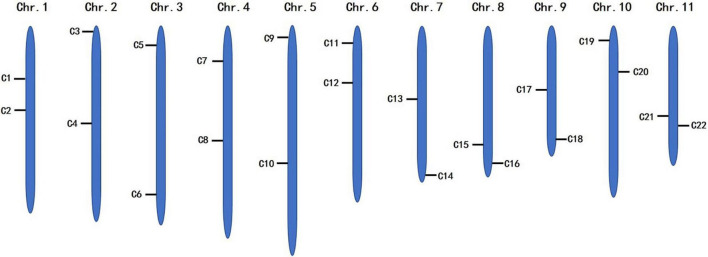
The distribution of 22 core SNP markers on 11 bottle gourd chromosomes.

**FIGURE 3:**
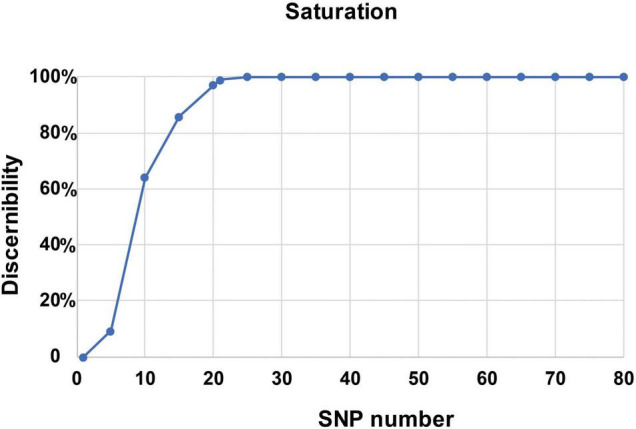
A saturation curve of the 22 core SNP markers identified in 206 bottle gourd germplasm collections.

**FIGURE 4:**
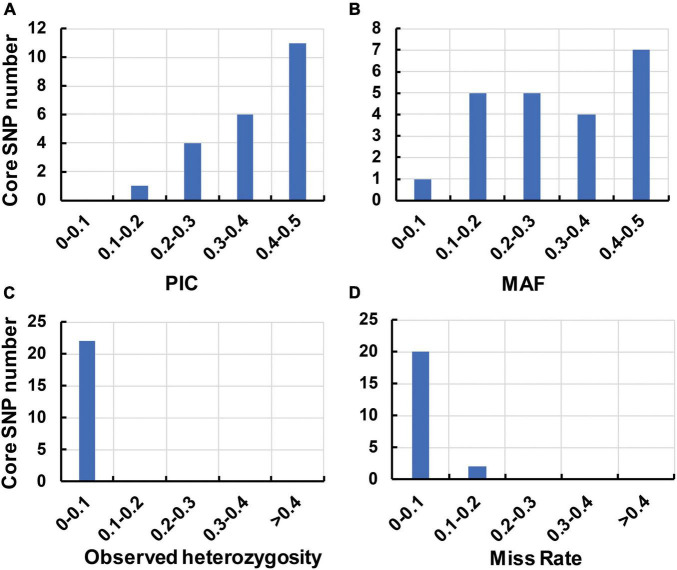
The genetic diversity indices of the core SNP marker set based on 206 bottle gourd germplasm collections. **(A)** The polymorphism information content (PIC). **(B)** The minor allele frequency (MAF). **(C)** The observed heterozygosity. **(D)** The rate of occurrence of missing values.

To identify the candidate core SNPs, the 206 bottle gourd germplasm collections were utilized for KASP assays with the 93 high-quality SNPs. A core SNP marker set was selected by considering the physical position, PIC, MAF, observed heterozygosity, and missing values among all 206 genotypes. The core marker set comprised 22 SNP markers, with two markers per chromosome (Figure 2 and Table 1). The saturation curve presented in Figure 3 shows that 22 core SNP markers could distinguish all 206 bottle gourds. The PIC for the 22 core SNP markers across all examined germplasm collections ranged from 0.137 to 0.499, with an average value of 0.390. For 17 of the 22 core SNP markers, we obtained PIC values >0.30 (Figure 4A and Supplementary Table 2), thereby indicating that the 22 core SNP markers were sufficiently polymorphic. The average MAF value for these 22 markers was 0.302 (Figure 4B and Supplementary Table 2). The observed heterozygosity of loci in the 206 bottle gourds loci was <0.10, with an average of 0.024 (Figure 4C and Supplementary Table 2). Missing values comprised <0.10 of data points in 90.9% (20/22) of the core SNP markers (Figure 4D and Supplementary Table 2). Details (marker name, chromosome, position, variation type, and primer sequences) of all 22 core SNP markers are listed in Table 1.

**TABLE 1:** KASP primer names, chromosomes, positions, variation types, and sequences for the 22 core SNP markers.

Name	Chromosome	Position	Variation type	Primer sequences
C1	1	8431159	A/T	F1: GAAGGTGACCAAGTTCATGCTGCTAAAGAGTTTAACTGGTTAATCTTAGATA F2: GAAGGTCGGAGTCAACGGATTGCTAAAGAGTTTAACTGGTTAATCTTAGATT R: CTAATGGACCTACAAATCATGAACTCCAA
C2	1	14051095	T/A	F1: GAAGGTGACCAAGTTCATGCTTCAATGTCCTGATCTTGTTGTCATCTT F2: GAAGGTCGGAGTCAACGGATTTCAATGTCCTGATCTTGTTGTCATCTA R: TTTCATATAACATGGACCTTGGATGGTTATA
C3	2	589455	T/C	F1: GAAGGTGACCAAGTTCATGCTTATATTAGGTTTAAATGCTACTTTGGTCCT F2: GAAGGTCGGAGTCAACGGATTTATTAGGTTTAAATGCTACTTTGGTCCC R: GGACCAAAGTGAACCAAAACCAAAAGTATA
C4	2	15914116	G/A	F1: GAAGGTGACCAAGTTCATGCTAAATTTTGTTAAACTCGTTTCCGTTCATAG F2: GAAGGTCGGAGTCAACGGATTGAAATTTTGTTAAACTCGTTTCCGTTCATAA R: TAACCTCAAAGTTCTAAACTCAAAATTATTCTTTA
C5	3	3421007	A/T	F1: GAAGGTGACCAAGTTCATGCTGTGGCCTCACCCACTATTTTTCAAA F2: GAAGGTCGGAGTCAACGGATTGTGGCCTCACCCACTATTTTTCAAT R: GTATTGTGTATTTTGTGTTATCTGATTGTTATATTT
C6	3	28241399	T/C	F1: GAAGGTGACCAAGTTCATGCTTAGTAGTCTTAGTGATCTCGAAGGAAT F2: GAAGGTCGGAGTCAACGGATTGTAGTCTTAGTGATCTCGAAGGAAC R: TTCGAGATCACTATGACTGCCATGATAT
C7	4	5104280	T/C	F1: GAAGGTGACCAAGTTCATGCTGAGACATGTGGCATTTTTTTAGTTT F2: GAAGGTCGGAGTCAACGGATTGAGACATGTGGCATTTTTTTAGTTC R: CCATGTCATTACAACGAAAGTCC
C8	4	20784251	C/A	F1: GAAGGTGACCAAGTTCATGCTCCGGACCTGTTCACTTCATCAC F2: GAAGGTCGGAGTCAACGGATTGCCGGACCTGTTCACTTCATCAA R: GATTCAGCTACGCCGCCGTCAA
C9	5	1706273	C/T	F1: GAAGGTGACCAAGTTCATGCTGAGAAGATCAATAGAAACCCC F2: GAAGGTCGGAGTCAACGGATTGAGAAGATCAATAGAAACCCT R: CCTGTGCCTGATGCTCATGTCC
C10	5	27139793	T/C	F1: GAAGGTGACCAAGTTCATGCTGTTCCCACTACCACTAGGCCAAT F2: GAAGGTCGGAGTCAACGGATTTTCCCACTACCACTAGGCCAAC R: AGTGTATTAAATTAAAGAAGCATTTAAACCATCAT
C11	6	1594854	A/G	F1: GAAGGTGACCAAGTTCATGCTGAGCTTAACTTGCTATGCACCTAGA F2: GAAGGTCGGAGTCAACGGATTAGCTTAACTTGCTATGCACCTAGG R: CCATTAAGAGGGAGTCTCACATCTAAAA
C12	6	5974943	G/A	F1: GAAGGTGACCAAGTTCATGCTGTACTATTGTCAATTATACATGCTGAGG F2: GAAGGTCGGAGTCAACGGATTGTACTATTGTCAATTATACATGCTGAGA R: GACCGACTCTCTCAACCATATCCAT
C13	7	11605465	T/C	F1: GAAGGTGACCAAGTTCATGCTTCGATGGTGTTCGTGATGAGACT F2: GAAGGTCGGAGTCAACGGATTCGATGGTGTTCGTGATGAGACC R: CATATTGCCCATGAGGTGAGGCTT
C14	7	23829758	T/C	F1: GAAGGTGACCAAGTTCATGCTGGATAGATGGGGATCAGCT F2: GAAGGTCGGAGTCAACGGATTGGATAGATGGGGATCAGCC R: AAAAACTTGCATTGCGAACTCC
C15	8	20000834	A/G	F1: GAAGGTGACCAAGTTCATGCTCCACTCTACCCACCCGAGGA F2: GAAGGTCGGAGTCAACGGATTCACTCTACCCACCCGAGGG R: GTAATGTTGTTGCTCATTCTTCGGCTTAAA
C16	8	22529614	T/C	F1: GAAGGTGACCAAGTTCATGCTGTGGACTGTTAATGTACCCATGTGAT F2: GAAGGTCGGAGTCAACGGATTTGGACTGTTAATGTACCCATGTGAC R: TAGAGCATCATATCAATCACAGGCCTAA
C17	9	10095406	T/C	F1: GAAGGTGACCAAGTTCATGCTTTGCAAATTCCTCCCAAATTGAGTAGT F2: GAAGGTCGGAGTCAACGGATTGCAAATTCCTCCCAAATTGAGTAGC R: CTAGGGTACTACTCATGATTCTATCTCTT
C18	9	18607803	A/G	F1: GAAGGTGACCAAGTTCATGCTTTGCATACTATCGATTGTAAGAAGGAAAAA F2: GAAGGTCGGAGTCAACGGATTGCATACTATCGATTGTAAGAAGGAAAAG R: CAACGCTCTTGCCAGTAATTCTTTGATT
C19	10	2343761	C/A	F1: GAAGGTGACCAAGTTCATGCTTCGTTGATGGGTGACGGTAAATTTC F2: GAAGGTCGGAGTCAACGGATTTATCGTTGATGGGTGACGGTAAATTTA R: GACCAAACACACATATTGTTAGATGATATAATAA
C20	10	4261004	T/C	F1: GAAGGTGACCAAGTTCATGCTCAGCTTATGTTTCCTGTTCTAGT F2: GAAGGTCGGAGTCAACGGATTCAGCTTATGTTTCCTGTTCTAGC R: AGAGAACTCAAGATCACCTCCCAAGT
C21	11	14865743	G/T	F1: GAAGGTGACCAAGTTCATGCTATAGTTTGATCTAGAATTGTTTGTAATAATTTG F2: GAAGGTCGGAGTCAACGGATTGATAGTTTGATCTAGAAATTGTTTGTAATATTT R: ACAAACATTAGAAACTTTTACAACTTACACACTT
C22	11	15431610	G/A	F1: GAAGGTGACCAAGTTCATGCTATTCTAATACTTTGAGAATACAAACTCTTTTTG F2: GAAGGTCGGAGTCAACGGATTATTCTAATACTTTGAGAATACAAACTCTTTTTA R: GCCAATGAAATAGAAATAATATATCACATGTAAAAT

### Polymorphism and Discriminatory Capacity of the Core Single-Nucleotide Polymorphism Set

We initially evaluated the polymorphism of the core SNP set using KASP assays in a MAGIC population, which had been constructed from eight genetically diverse elite parents and consisted of 377 recombinant inbred lines. Data obtained for individuals from these 377 lines were used to calculate the PIC, MAF, observed heterozygosity, and missing values for each core SNP marker. The PIC values of the 22 core SNP markers across the 377 individuals ranged from 0.12 to 0.50, with an average of 0.38. Sixteen SNP markers had PIC values >0.3, and the MAF values ranged from 0.07 to 0.45, with an average of 0.30. The observed heterozygosity for each core SNP marker was ≤0.10, with an average of 0.04. For all core SNP markers, the missing values comprised ≤0.06 of the data points (Table 2). Collectively, these results indicate that the core SNP markers were highly polymorphic.

**TABLE 2:** Genetic diversity indices for the 22 core SNP markers based on the multiparent advanced generation inter-cross (MAGIC) population.

Marker	PIC	MAF	Heterozygosity	Missing value
C1	0.50	0.45	0.05	0.02
C2	0.24	0.14	0.03	0.06
C3	0.50	0.45	0.06	0.01
C4	0.46	0.35	0.06	0.02
C5	0.49	0.44	0.05	0.01
C6	0.22	0.12	0.02	0.01
C7	0.46	0.35	0.10	0.01
C8	0.45	0.34	0.06	0.00
C9	0.32	0.20	0.01	0.06
C10	0.29	0.18	0.06	0.01
C11	0.49	0.42	0.06	0.02
C12	0.33	0.21	0.01	0.01
C13	0.39	0.27	0.04	0.02
C14	0.48	0.39	0.08	0.03
C15	0.37	0.24	0.06	0.01
C16	0.47	0.38	0.05	0.02
C17	0.50	0.47	0.01	0.01
C18	0.50	0.46	0.00	0.00
C19	0.28	0.17	0.07	0.02
C20	0.49	0.42	0.09	0.02
C21	0.12	0.07	0.01	0.06
C22	0.14	0.07	0.00	0.00

*PIC and MAF indicate polymorphism information content and minor allele frequency, respectively.*

**FIGURE 5:**
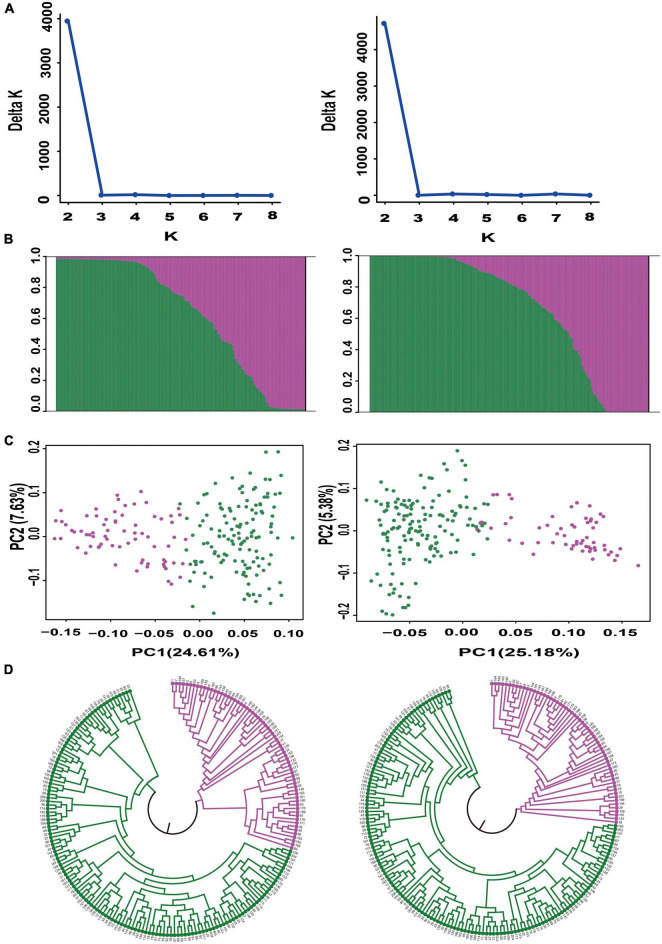
Population structure analysis of 206 bottle gourd germplasm collections using the core SNP marker set (left) and 93 high-quality SNPs (right). **(A)** Delta *K* values. **(B)** Population structure of the germplasm collection inferred at *K* = 2. The *Y*-axis quantifies cluster membership, and the *X*-axis lists the different germplasm collections. **(C)** Principal component analysis (PCA) of the 206 bottle gourd germplasm collections. **(D)** Neighbor-joining tree showing a dendrogram of the 206 bottle gourd germplasm collections. Pink and green color indicated two different subpopulations.

We further evaluated the discriminatory power of the core SNP set by analyzing the genetic structure of the 206 bottle gourd germplasm collections using the core SNP set and the aforementioned 93 high-quality SNPs. Assessment of the relationships among the 206 bottle gourd germplasm collections using STRUCTURE showed that the best Δ*K* value was 2, which divided the 206 bottle gourd germplasm collections into two groups when using either the 93 high-quality SNPs or the core SNP marker set (Figures 5A,B). The clustering results obtained for the core SNP marker set and 93 high-quality SNPs differed only with respect to 14 of the collections (Supplementary Table 3). PCA and the UPGMA dendrogram exhibited similar results when using the two different SNP marker sets (Figures 5C,D). The aforementioned results thus indicate that the core SNP marker set had strong representativeness and discriminatory power equal to the 93 well-selected high-quality SNPs.

### Applications of the Core Single-Nucleotide Polymorphism Marker Set

Molecular fingerprinting of bottle gourd germplasm collections or commercial cultivars is a potential practical application of the newly developed core SNP marker set. In this study, 206 bottle gourd germplasm collections were fingerprinted using the 22 core SNP markers (Table 3 and Supplementary Table 4), which highlighted the efficiency and accuracy of genotype discrimination using the 22 core SNP markers. Furthermore, representative cultivars (hybrids) currently on the market, and with unique barcodes and QR codes, were genotyped using the 22 core SNP markers (Table 4), which indicates the potential contribution of these markers in resolving intellectual property disputes caused by the use of the same name for different species or different names for the same species. Moreover, the SNP fingerprints provided a precise, rapid, convenient, and cost-effective KASP genotyping procedure for determining bottle gourd seed purity.

**TABLE 3:** The collection site, fruit shape, and core SNP fingerprint information of a proportion of the 206 bottle gourd germplasm collections.

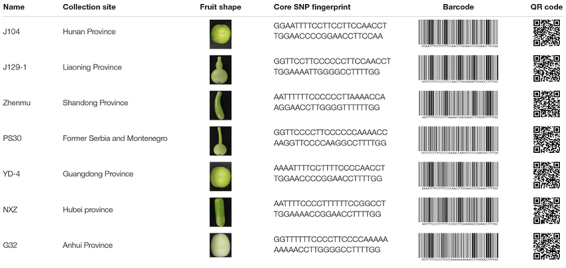

**TABLE 4:** The photos, descriptions, and core SNP fingerprint information of representative cultivars in the market.

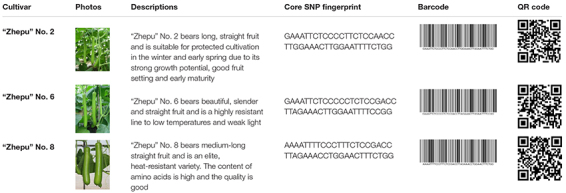

### Development of a Core Collection

**FIGURE 6:**
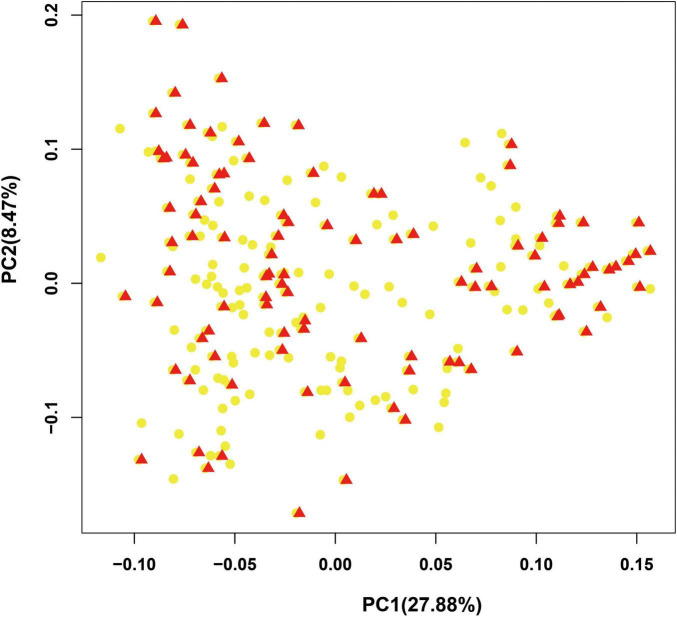
Evaluation of the bottle gourd core collection by principal component analysis (PCA). Red triangles: accessions in the core collection; yellow dots: accessions in the original collection.

To provide a subset of representative germplasm collections for the selection of parents in hybrid combinations in bottle gourd breeding or related basic studies, we further developed a core collection of bottle gourd germplasm collections using the 22 core SNP markers and Core Hunter software (Thachuk et al., 2009). The core collection included 102 representative bottle gourd germplasm collections, which captured approximately 50% of the total number of germplasm collections with 100% allele coverage (marked in red in Supplementary Table 1). Two indices were used to measure the average genetic distance of the core collection population: the modified Rogers distance (MR) and Cavalli-Sforza and Edwards distance (CE), with the values of 0.4424 and 0.44427, respectively. Furthermore, three genetic diversity indices were calculated: Shannon’s diversity index (SH), expected heterozygous (HE), and PIC with the values of 2.9714, 0.425644, and 0.332121, respectively (Table 5). Additionally, the results obtained from PCA analysis of the 102 germplasm collections in the core collection were to a large extent consistent with those obtained for the original collection (Figure 6). Therefore, the core collection is representative of the genetic diversity of the original collection. Considering suitable size, geographical distribution, phenotype, and unique agronomic traits, an additional 48 germplasm collections were added to the initial core collection, giving a final core collection containing 150 bottle gourd germplasm collections.

**TABLE 5:** Evaluation of the genetic diversity of core bottle gourd germplasm collections.

Initial collection	Core collection	MR	CE	SH	HE	NE	PIC	CV
206	102	0.44	0.44	2.97	0.42	1	0.33	100%

*MR, CE, SH, HE, NE, PIC, and CV indicate modified Rogers distance, Cavalli-Sforza and Edwards distance, Shannon’s diversity index, expected heterozygosity, number of effective alleles, polymorphism information content, and coverage of alleles, respectively.*

## Discussion

A range of DNA molecular markers, including RAPDs, SSRs, InDels, and SNPs, have been used for germplasm characterization (Chen and Sullivan, 2003; Lv et al., 2012; Hao et al., 2016; Yang et al., 2019). Among these, SNPs, with their unique characteristics of wide distribution, high density, and good stability, combined with a cost-effective, user-friendly SNP genotyping platform (KASP), have become a popular marker type for germplasm characterization and cultivar fingerprinting (Allen et al., 2010; Cortés et al., 2011; Su et al., 2018; Wang et al., 2018; Li et al., 2019; Wu et al., 2021). However, although RAPD, SSR, and InDel markers, which involve complex processes, would be superseded by the development of SNP molecular markers, which are suitable for large-scale high-throughput screening of multiple samples and sites, SNPs combined with KASP are yet to be utilized for large-scale germplasm characterization in bottle gourd.

In this study, using the high-throughput SNP genotype platform, we selected and developed a core set of SNP markers from an initial 1,100 SNPs identified by the re-sequencing of 20 bottle gourd representatives. The representativeness and discriminatory power of the core SNP marker set were evaluated using 206 bottle gourd germplasm collections and a MAGIC population (Figure 4 and Table 2). We found that the core SNP marker set had strong representativeness and discriminatory power equal to that of 93 high-quality SNPs (Figure 5). The use of fewer markers is more convenient for identifying varieties or fingerprinting cultivars than using large numbers of markers. Different subsets of markers show different identification rates; for example, in cultivated pumpkin, subsets of 24 and 12 SNP markers identified only 24.2% and 4.9% accessions, respectively (Nguyen et al., 2020). The core SNPs were identified to represent the greatest possible genetic diversity using the minimum number of SNPs. For example, in non-heading Chinese cabbage, 50 core SNPs were found to provide adequate information for genetic identification (Li et al., 2019). A core set of 16 SSRs has been shown to be sufficient to identify 382 cucumber varieties and establish DNA fingerprints (Yang et al., 2019). Similarly, in cowpea, 50 informative core SNPs were shown to be strongly representative of the 51,128 SNPs available to analyze genetic dissimilarity in this species (Wu et al., 2021). In this study, a saturation curve revealed 22 abundant polymorphisms, and uniformly distributed core SNP markers distinguished 100% of 206 bottle gourds (Figure 3), thereby indicating that these 22 core SNP markers were sufficiently discriminatory for the identification of bottle gourd germplasm.

The verification of seed authenticity and purity is of particular importance for seed producers and farmers (Gao et al., 2012). Similar genetic backgrounds often make it difficult to morphologically identify species using low-efficiency and time-consuming field planting. Moreover, morphological characteristics are often influenced by the environment and are therefore not suitable for the current rapid inspection demands (Tian et al., 2015). A potential application of the core SNP marker set is the molecular fingerprinting of bottle gourd germplasm collections or commercial cultivars to preserve and utilize germplasm collections and determine the authenticity and purity of cultivars. We fingerprinted 206 bottle gourd germplasm collections and representative bottle gourd commercial hybrids with unique barcodes and QR codes (Tables 3, 4) and developed optional primers for determining bottle gourd seed purity. Owing to the biallelic nature of SNP markers, each marker can distinguish three individuals. The maximum number of individuals distinguishable using the set of 22 core SNP markers selected in this study is, in theory, 3^22^ = 31,381,059,609. Therefore, it is feasible to construct a fingerprint database of bottle gourd germplasm collections or main commercial varieties using the 22 core SNP markers.

The construction of a core collection will substantially improve the efficiency of germplasm collection management and utilization. Core collections established using molecular markers are not readily affected by environmental or other external factors, and hence, several core collections have been constructed using various DNA molecular markers (Balfourier et al., 2007; Zhang et al., 2011; Muñoz-Amatriaín et al., 2014). In this study, we developed a core collection of 102 accessions that represent 100% of the bottle gourd collections in China (Table 5 and Figure 6). Previous studies have revealed weak population stratification and low diversity in bottle gourd germplasm collections, which are generally independent of the site of collection (Yetişir et al., 2008; Xu et al., 2011, 2014, 2021). Taking into consideration the factors of suitable size, phenotype, and unique agronomic traits, we augmented our original core collection with an additional 48 inbred lines, thereby establishing a final core collection containing 150 bottle gourd inbred lines. To the best of our knowledge, this study represents the first effort to preserve and utilize germplasm collections of bottle gourd, and the core collection thus developed will contribute substantially to future bottle gourd breeding and research. Accordingly, we believe that the genomes of the accessions selected for the core collection should be re-sequenced to provide a valuable resource for future breeding and scientific studies.

In summary, based on the re-sequenced genomic data from 20 bottle gourd germplasm collections, we identified and validated a core set of 22 representative SNPs, which exhibited abundant polymorphisms and were evenly distributed throughout the bottle gourd genome. Using this core SNP marker set, we assessed the genetic diversity and population structure of bottle gourd collections, fingerprinted bottle gourd germplasm collections and commercial cultivars, performed an optimized procedure for seed authentication, and developed an accessible core population. Our findings will provide a valuable basis for the future preservation and utilization of bottle gourd germplasm collections and also contribute to cultivar identification, which will enable the resolution of commercial disputes and protect the rights of breeders.

## Data Availability Statement

The original contributions presented in the study are included in the article/Supplementary Material, further inquiries can be directed to the corresponding author.

## Author Contributions

YW and GL conceived the research. YW, XaW, YL, ZF, ZM, JW, XnW, BW, and ZL performed the experiments. XaW provided the mutant material. YL, ZF, ZM, JW, and XnW provided technical assistance. YW analyzed the data and wrote the manuscript. All authors contributed to the article and approved the submitted version.

## Conflict of Interest

The authors declare that the research was conducted in the absence of any commercial or financial relationships that could be construed as a potential conflict of interest.

## Publisher’s Note

All claims expressed in this article are solely those of the authors and do not necessarily represent those of their affiliated organizations, or those of the publisher, the editors and the reviewers. Any product that may be evaluated in this article, or claim that may be made by its manufacturer, is not guaranteed or endorsed by the publisher.

## Footnotes

1
http://statgen.ncsu.edu/powermarker/

2
http://barcode.tec-it.com/barcode-generator.aspx

## Supplementary Material

Supplementary Figure 1 Construction process of the MAGIC population used in this study. The characteristics of parental lines were: J077: corbel, green with white spots; J120: tubby, strong umami taste and high amino acid content; G32: round; Yaohulu: hulu (double-gourd); J104: round, strong umami taste and high amino acid content; J098: middle straight; J083: corbel, resistance to powdery mildew; J7: slender straight, strong resistance.
