# Supplementary figures and images for: Identification and Validation of a Core Single-Nucleotide Polymorphism Marker Set for Genetic Diversity Assessment, Fingerprinting Identification, and Core Collection Development in Bottle Gourd
Source: Front Plant Sci. 2021 Nov 18;12:747940. doi: 10.3389/fpls.2021.747940 (PMC8636714; doi:10.3389/fpls.2021.747940)

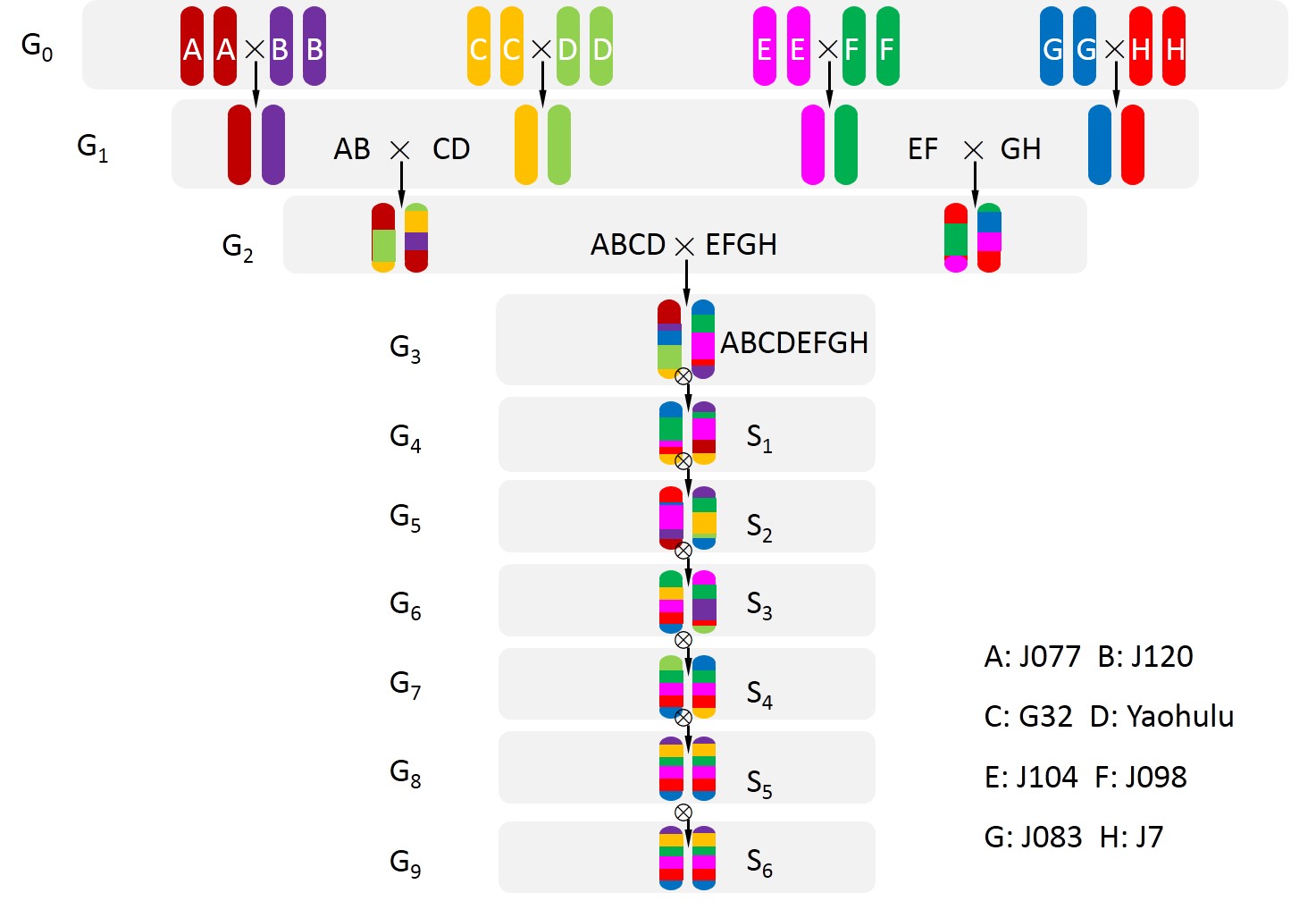

Supplement: Supplementary Figure 1 — Construction process of the MAGIC population used in this study. The characteristics of parental lines were: J077: corbel, green with white spots; J120: tubby, strong umami taste and high amino acid content; G32: round; Yaohulu: hulu (double-gourd); J104: round, strong umami taste and high amino acid content; J098: middle straight; J083: corbel, resistance to powdery mildew; J7: slender straight, strong resistance. [file Image_1.JPEG]
